# Baseline endocrine factors influencing live birth outcomes in Chinese infertile women undergoing their first fresh IVF cycle: A multistate model-based cohort study

**DOI:** 10.1371/journal.pone.0349394

**Published:** 2026-06-05

**Authors:** Yajun Dong, Zhonghua Ai, Shuhong Luo, Yue Yang, Yan Huang, Dan Zhang, Yan Jia, Hongxia Ye

**Affiliations:** 1 Department of Reproductive Immunology, Sichuan Jinxin Xi’nan Women’s and Children’s Hospital, Chengdu, Sichuan, China; 2 Department of Epidemiology, School of Public Health, Sun Yat-sen University, Guangzhou, China; King Saud University / Zagazig University, EGYPT

## Abstract

Conventional in vitro fertilization (IVF) outcome prediction is limited by static, single-endpoint analyses. We aimed to overcome this by using a multistate model to dissect the stage-specific and, crucially, the non-linear influence of endocrine factors across the entire pregnancy continuum in a large-scale cohort. We applied multistate regression models to a large cohort of 12,674 women undergoing their first fresh IVF cycle. This advanced method allowed us to analyze three sequential transitions (from infertility to biochemical pregnancy, from biochemical pregnancy to clinical pregnancy, and ultimately to live birth) and test for non-linear effects of baseline hormones, including anti-Müllerian hormone (AMH), luteinizing hormone (LH), and antral follicle count (AFC), on the hazard of success at each stage. The principal finding was a significant non-linear relationship between baseline AMH, LH, and AFC and pregnancy success (P < 0.05 for non-linearity). This directly challenges the “higher is better” paradigm, revealing that optimal hormonal “windows”, not just maximum levels, are linked to clinical success. The multistate model further distinguished AMH and LH as robust predictors across all stages, while AFC’s predictive power was confined to achieving initial pregnancy. The predictive value of baseline hormones in IVF is fundamentally non-linear. Our use of a multistate model demonstrates that while AMH and LH are consistent predictors for the entire pregnancy journey, their clinical interpretation must shift from a linear scale to identifying optimal ranges. This finding provides a more precise scientific basis to personalize ART treatment and improve live birth rates.

## Introduction

Infertility is a global health concern, and Assisted Reproductive Technology (ART), particularly in vitro fertilization (IVF), has become the primary treatment modality [1–4].

In recent years, the rise of the elective freeze-all (eFET) strategy has sparked a widespread debate over its superiority compared to fresh embryo transfer [5,6], While eFET may improve live birth rates (LBR) in certain high-responder populations and significantly reduce the risk of ovarian hyperstimulation syndrome (OHSS), its advantages are not universal and may be associated with an increased risk of pre-eclampsia and higher treatment costs [7–9]. Therefore, for patients undergoing their first IVF cycle, fresh embryo transfer remains a crucial and indispensable treatment strategy, especially when comprehensively considering clinical efficacy, patient burden, and potential risks. Accurately identifying key factors influencing LBR in fresh embryo transfer cycles is of significant importance for guiding clinical decision-making, optimizing treatment protocols, and improving first-attempt success rates.

However, previous studies on predictors of ART outcomes have often focused on single time-point outcomes (e.g., live birth) [10,11]. They have less frequently systematically evaluated the dynamic role of basal endocrine hormones throughout the continuous transition of pregnancy-from embryo implantation to live birth and whether these effects exhibit non-linear characteristics.

Therefore, this study aims to utilize an innovative statistical approach, the multi-state model, in a large cohort of Chinese infertile patients, to thoroughly investigate the association between various basal endocrine hormones and the continuous transition from infertility to live birth following the first fresh embryo transfer cycle. We specifically focus on whether these associations exhibit non-linear characteristics and hypothesize that the predictive value of certain key hormones (such as AMH and LH) will persist throughout the entire pregnancy continuum, up to live birth.

## Methods

### Study design and participants

This retrospective cohort study was conducted at the Jinxin Xi’nan Women’s and Children’s Hospital in Sichuan, China. We included infertile patients who underwent their first fresh-cycle in vitro fertilization (IVF) or intracytoplasmic sperm injection (ICSI) treatment between January 2019 and July 2022. An initial cohort of 13,157 participants was identified from the hospital’s electronic medical records system.

### Inclusion and exclusion criteria

**Fig 1:**
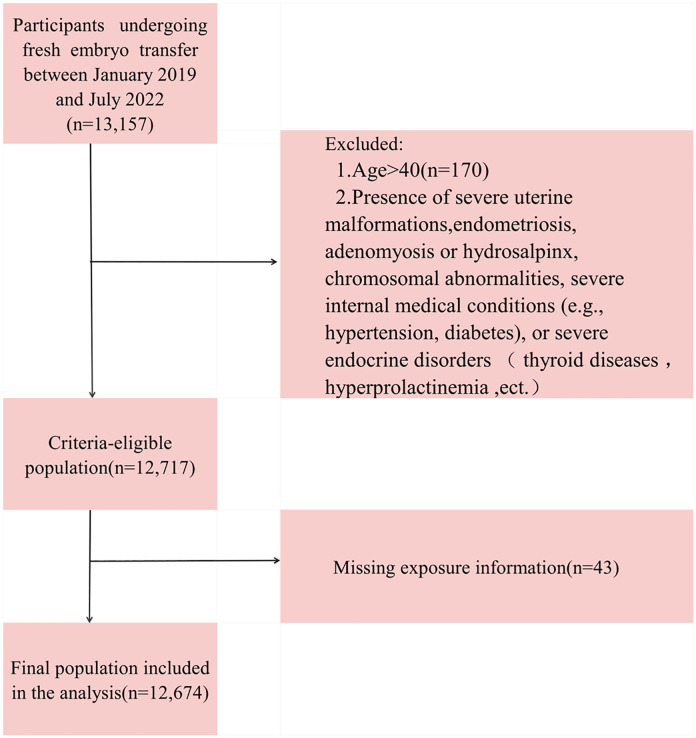
Patient inclusion flowchart.

The primary inclusion criterion was women undergoing their first fresh embryo transfer cycle. Participants were excluded based on the following criteria, as detailed in the study flowchart (Fig 1): Age > 40 years (n = 170), to focus on the core reproductive population and minimize age-related confounding factors. Presence of severe uterine malformations, endometriosis, adenomyosis or hydrosalpinx, chromosomal abnormalities, severe internal medical conditions (e.g., hypertension, diabetes), or severe endocrine disorders such as thyroid diseases，hyperprolactinemia, ect. (n = 270). Missing essential baseline endocrine or outcome information (n = 43). After applying these criteria, a final cohort of 12,674 participants was included in the analysis.

### Data collection and variables

All data for this study were retrospectively collected from the electronic medical records system of our hospital. A standardized data extraction form was used to collect information on: Baseline characteristics: weight height, age, ethnicity, region of residence education level, and male smoking status, etc. Clinical and laboratory parameters: Baseline Ovarian Reserve and Endocrine Profile (e.g., AMH, E2, P, LH, FSH, TSH, AFC) Ovarian Stimulation and Cycle Parameters, Embryological Data and Embryo Transfer Details，Pregnancy Outcomes, etc.

### Data quality control

To ensure data accuracy, all data were extracted independently by two trained physicians (Y. Huang and Y. Yang), with discrepancies resolved by a senior investigator (Y. Jia). A random 10% of the data was further cross-checked against original records for verification.

### Ethical considerations

This retrospective cohort study was conducted in accordance with the Declaration of Helsinki and received full approval from the Reproductive Ethics Committee of Chengdu Xi’nan Women’s and Children’s Hospital [Approval No. (2023) Repro. Ethics Comm. Approval No. (09)]. Specifically, the ethics committee waived the requirement for individual patient informed consent. This waiver was granted because the study involved the secondary analysis of a large-scale, fully anonymized clinical dataset, where all patient-identifying information had been removed prior to access by the research team. This process ensures that it is not feasible to re-identify patients and that the use of their data poses no risk to their privacy. The data were accessed for research purposes from 01/05/2023 to 31/08/2023.

### Ovarian stimulation and IVF procedures

All patients included in this study underwent individualized controlled ovarian stimulation (COS) protocols. These protocols were formulated and administered by senior reproductive physicians in our center, strictly adhering to the evidence-based recommendations outlined in the ESHRE Guideline for Ovarian Stimulation for IVF/ICSI and the expert consensus guidelines from the Reproductive Medicine Professional Committee of the Chinese Medical Doctor Association [12].

The choice of protocol and the dosage of gonadotropins were determined by the attending physician based on a comprehensive assessment of patient characteristics, primarily including age, body mass index (BMI), and ovarian reserve markers such as anti-Müllerian hormone (AMH) and antral follicle count (AFC)，Follicle-Stimulating Hormone (FSH),luteinizing hormone (LH), estradiol (E2),progesterone (P). The most frequently utilized stimulation protocols were the gonadotropin-releasing hormone (GnRH) agonist long protocol, the luteal phase GnRH agonist protocol, and the GnRH antagonist protocol.

Follicular development was continuously monitored using transvaginal ultrasound and serum hormone level measurements. When at least 2–3 dominant follicles reached an average diameter of 18 mm, human chorionic gonadotropin (hCG) or a GnRH agonist was administered to induce final oocyte maturation. Oocyte retrieval was performed 34–36 hours post-trigger injection via transvaginal ultrasound-guided aspiration.

Fertilization was achieved using conventional in vitro fertilization (IVF) or intracytoplasmic sperm injection (ICSI), with the specific method selected based on semen analysis parameters and the couple’s prior fertilization history. Embryos were cultured in vitro and their quality was assessed sequentially. On day 3 post-oocyte retrieval, cleavage-stage embryos were evaluated based on their morphology, including the number of blastomeres, fragmentation rate, and blastomere symmetry. For patients scheduled for a day 5 transfer or for embryos selected for extended culture, development was continued to the blastocyst stage. The quality of all resulting blastocysts was subsequently assessed on day 5 or day 6 using the Gardner grading system, which evaluates the degree of blastocoel expansion, the inner cell mass (ICM), and the trophectoderm (TE) [13].

For this study cohort, fresh embryo transfer was performed on day 3 or day 5. Typically, 1–2 top-quality embryos were selected for transfer into the uterine cavity under ultrasound guidance. Patients were considered suitable for fresh transfer if they had no significant risk of ovarian hyperstimulation syndrome (OHSS), good endometrial receptivity, and no premature elevation of serum progesterone levels. All patients received luteal phase support starting from the day of oocyte retrieval, typically with vaginal and/or oral progesterone preparations, to support embryo implantation and early pregnancy. Any remaining good-quality usable embryos were cryopreserved using vitrification for future use.

### Outcome definitions

Biochemical Pregnancy: Defined as a serum β-human chorionic gonadotropin (β-hCG) level ≥30 IU/L at 12–14 days post-embryo transfer. Clinical Pregnancy: Defined as the presence of an intrauterine gestational sac visualized by transvaginal ultrasound 4–5 weeks post-embryo transfer. Live Birth: Defined as the delivery of at least one live infant after 28 completed weeks of gestation.

### Assessment of covariates

We developed a directed acyclic graph (DAG) to identify variables for adjustment [14]. We first included sociodemographic and physiological factors, including age, ethnicity, education level (high school and below, specialist or bachelor’s degree, master’s degree and above), body mass index (BMI), total bilirubin, direct bilirubin, blood glucose of female, male smoking status (No/Yes).

Relevant reproductive factors were also considered, including the high-quality embryos at the cleavage stage, the number of high-quality embryos transferred, endometrial thickness, infertility type (primary or secondary), treatment programmes (long programmes, short programmes, other programmes) female health status (healthy, fair, illness). These factors were included to account for their potential influence on reproductive outcomes [12,15,16].

### Statistical analysis

In describing the characteristics of the participants included in the study, we used the mean and standard deviation for statistical descriptions for continuous variables that conformed to a normal distribution, and the median and interquartile range (IQR) for continuous variables with a skewed distribution. For categorical variables, we reported frequencies and proportions.

**Fig 2:**
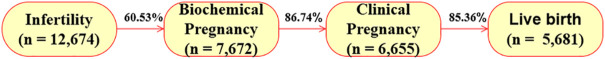
The progression trajectory of infertile patients from biochemical pregnancy to clinical pregnancy and finally to live birth (N = 12,674).

Given that this study aims to analyze the continuous multistage process from infertility to live birth, traditional single-endpoint survival analysis (such as the Cox model targeting only live birth) cannot test the differential effects of reproductive parameters on transitions across different stages within a unified framework [17]. Therefore, we adopted a stepwise, complementary modeling strategy. Firstly, we used the Cox proportional risk model to estimate the association between reproductive endocrine hormones and different state transitions, and then further analyses were performed based on a multistate regression model using a clock-forward approach as a time scale (Fig 2). The multistate model consisted of three states (positive biochemical pregnancy, positive clinical pregnancy and live birth) with three transitions between these states: a) transitions from infertility to positive biochemical pregnancy, b) transitions from positive biochemical pregnancy to positive clinical pregnancy and c) transitions from positive clinical pregnancy to live birth. This model enabled simultaneous analysis of all transition pathways and examination of the differential effects of reproductive parameters across these pathways. Restricted cubic spline modelling was used to assess potential non-linear relationships between reproductive endocrine hormones and the progression of the three states at the 10th, 50th and 90th percentiles in 3-node increments [18].

### Sensitivity analysis

Several sensitivity analyses were conducted to check the robustness of our results. First, taking into account the effect of excessive years of infertility on the results, we excluded 294 participants who had been infertile for more than 10 years, and 12,380 participants were included in this replicated analysis. Second, to assess the robustness of the findings in a potentially susceptible population, we performed subgroup analyses according to whether the pregnant women had a normal BMI. These combined analytical strategies provided us with a comprehensive and robust perspective to assess the association between relevant hormones and assisted conception outcomes.

All statistical tests were two-sided and p < 0.05 was used as the criterion for determining statistical significance. The multi-state model was constructed with the help of the mState package of R software (version 4.4.1, provided by the R development core team).

## Results

### Participant characteristics

Characteristics of the study participants are shown in Table 1. Among 12,674 infertile participants with IVF-ET, 7672 (60.53%) infertile patients developed a positive biochemical pregnancy, of which 6655 (86.74%) further developed a positive clinical pregnancy, and ultimately, 5681 (85.36%) clinically positive patients had a successful live birth. The mean age of successful live births was 30.48 years (with a standard deviation of 3.77 years), of which 5262 (92.60%) were Han Chinese, 2952 (52.00%) patients had high school and below education, and 3317 (58.40%) were treated with long programmes.

**Table 1:** Baseline characteristics of 12,674 patients, grouped by infertility without progression, infertility to biochemical pregnancy, biochemical pregnancy to clinical pregnancy, and clinical pregnancy to live birth.

Characteristics	Infertility without progress (n = 5002)	Infertility→ Biochemical pregnancy (n = 7672)	Biochemical pregnancy→ Clinical Pregnancy (n = 6655)	Clinical pregnancy→ Live birth (n = 5681)	*p*-Value
**Female age (years),** **mean (SD)**	31.30 (4.36)	30.64(3.88)	30.63 (3.88)	30.48 (3.77)	< 0.001
**Female ethnicity, n (%)**					0.006
Han ethnicity	4554 (91.0)	7082 (92.30)	6151 (92.40)	5262 (92.60)	
ethnic minority	448 (9.0)	572 (7.70)	504 (7.60)	419 (7.40)	
**Female education level, n (%)**					< 0.001
High school and below	2750 (55.0)	4023 (52.40)	3477 (52.20)	2952 (52.00)	
Specialist or bachelor’s degree	2137 (42.70)	3024 (44.60)	2985 (44.90)	2566 (45.10)	
Master’s degree and above	115 (2.30)	225 (3.0)	193 (2.90)	163 (2.90)	
**Male smoking status, n (%)**					0.996
No	4806 (96.10)	7375 (96.10)	6296 (96.10)	5459 (96.10)	
Yes	196 (3.90)	297 (3.90)	259 (3.90)	222 (3.90)	
**Body mass index (kg/m** ^**2**^ **), mean (SD)**	22.20 (3.12)	22.18 (3.10)	22.19 (3.09)	22.14 (3.07)	< 0.001
**Total Bilirubin (μmol/L), mean (SD)**	11.47 (4.66)	11.59 (4.82)	11.60 (4.86)	11.67 (4.91)	< 0.001
**Direct Bilirubin (μmol/L), mean (SD)**	2.56 (1.19)	2.57 (1.33)	2.58 (1.31)	2.60 (1.33)	< 0.001
**Blood glucose (mmol/L),mean (SD)**	5.21 (0.57)	5.22 (0.58)	5.23 (0.57)	5.23 (0.58)	< 0.001
**High-quality embryos at the cleavage stage** **(IQR)**	1 (3.00)	2 (3.00)	2 (3.00)	2 (3.00)	< 0.001
**Number of high-quality embryos transferred,** **median (IQR)**	1 (1.00)	1 (1.00)	1 (1.00)	1（1.00)	< 0.001
**Types of infertility, n (%)**					0.410
Primary infertility	2330 (46.60)	3651 (47.60)	3173 (47.70)	2738 (48.20)	
Secondary infertility	2672 (53.40)	4021 (52.40)	3482 (52.30)	2943 (51.80)	
**Endometrial thickness (mm), mean (SD)**	10.56 (1.88)	10.59 (1.95)	10.59 (1.94)	10.60 (1.93)	< 0.001
**AMH (ng/mL), median (SD)**	3.33 (3.22)	4.13 (2.19)	4.22 (2.17)	4.34(2.13)	< 0.001
**FSH (mIU/mL),** **median (SD)**	7.80 (2.30)	7.71 (2.22)	7.73 (2.24)	7.74 (2.26)	< 0.001
**AFC, median (IQR)**	13 (9.0)	17 (10.0)	18 (9.0)	18 (9.0)	< 0.001
**LH (miu/ml), mean (SD)**	4.35 (3.17)	6.34 (3.28)	6.62 (3.19)	7.01 (3.04)	< 0.001
**E2 (pg/ml), median (SD)**	47.79 (56.07)	45.94 (53.52)	46.18 (56.81)	46.02(52.46)	< 0.001
**P (ng/ml),** **median (SD)**	0.60 (0.34)	0.62 (0.36)	0.61 (0.34)	0.61 (0.34)	< 0.001
**TSH (uIU/mL), median (IQR)**	2.18 (1.54)	2.22 (1.53)	2.21 (1.52)	2.22 (2.47)	< 0.001
**Treatment programmes, n (%)**					< 0.001
Long programmes	2603 (52.0)	4393 (57.30)	3828 (57.50)	3317 (58.40)	
Short programmes	2311 (46.20)	3152 (41.10)	2721 (40.90)	2268 (39.90)	
Other programmes	88 (1.80)	127((1.60)	106 (1.60)	96 (1.70)	
**Female health status, n (%)**					0.980
Healthy	3737 (74.70)	5759 (75.10)	5006 (75.20)	4268 (75.10)	
Fair	309 (6.20)	445 (5.80)	384 (5.80)	332 (5.80)	
Illness	956 (19.10)	1468 (19.10)	1265 (19.0)	1081 (19.10)	

Note: Continuous variables were tested by analysis of variance and rank sum test, and categorical variables were tested by chi-square test. SD, standard deviation; IQR, interquartile range. AMH, Anti-mullerian hormone; AFC, Antral Follicle Count; FSH, Follicle Stimulating Hormone; LH, Luteinizing Hormone; E2, Estradiol; P, Progesterone; TSH, Thyroid-Stimulating Hormone.

### Using Cox regression to analyze the relationship between reproductive endocrine hormones and progression to assisted conception outcomes in infertile patients

In the multivariate Cox regression analysis, the results showed that the increase in reproductive endocrine hormones was significantly associated with three key transitions in treatment progression (see Table 2). Specifically, increases in AMH and LH were significantly associated with the transition from infertility to positive biochemical pregnancy, from positive biochemical pregnancy to positive clinical pregnancy, and from positive clinical pregnancy to successful live birth. AFC was only associated with the first two transitions, while E2 was associated only with the transition from positive biochemical pregnancy to positive clinical pregnancy. For example, a one-unit increase in AMH was positively correlated with the transition from infertility to positive biochemical pregnancy (HR = 1.030, 95% CI: 1.020, 1.040), from positive biochemical pregnancy to positive clinical pregnancy (HR = 1.035, 95% CI: 1.024, 1.046), and from positive clinical pregnancy to live birth (HR = 1.014, 95% CI: 1.001, 1.027). Additionally, a one-unit increase in AFC was positively correlated with the transition from infertility to positive biochemical pregnancy (HR = 1.015, 95% CI: 1.012, 1.018) and from positive biochemical pregnancy to positive clinical pregnancy (HR = 1.020, 95% CI: 1.017, 1.023).

**Table 2:** Hazard ratios (95% CI) associated with each 1-unit increase in reproductive endocrine hormones with biochemical pregnancy, clinical pregnancy, and subsequent live birth, calculated using a Cox model (N = 12,674).

Model	AMH	AFC	FSH	LH	E2	P	TSH
Model 1^a^							
Infertility → Biochemical Pregnancy	1.032 (1.022, 1.041)	1.016 (1.013, 1.019)	0.994 (0.984, 1.004)	1.030 (1.026, 1.034)	0.999 (0.999, 1.001)	1.018 (0.958, 1.081)	1.001 (0.999, 1.001)
Biochemical Pregnancy → Clinical Pregnancy	1.035 (1.024, 1.046)	1.020 (1.017, 1.023)	1.001 (0.991, 1.012)	1.038 (1.034, 1.042)	1.001 (1.001, 1.002)	0.984 (0.922, 1.051)	1.001 (0.999, 1.001)
Clinical Pregnancy → Live births	1.016 (1.003, 1.028)	1.002 (0.998, 1.006)	1.002 (0.991, 1.014)	1.009 (1.001, 1.017)	1.001 (0.999, 1.001)	1.034 (0.959, 1.114)	1.001 (0.999, 1.001)
Model 2^b^							
Infertility → Biochemical Pregnancy	1.030 (1.020, 1.040)	1.015 (1.012, 1.018)	0.996 (0.985, 1.006)	1.030 (1.026, 1.034)	0.999 (0.999, 1.001)	1.026 (0.965, 1.090)	1.001 (0.999, 1.001)
Biochemical Pregnancy → Clinical Pregnancy	1.035 (1.024, 1.046)	1.020 (1.017, 1.023)	1.002 (0.991, 1.013)	1.004 (1.001, 1.008)	1.001 (1.001, 1.002)	0.984 (0.922, 1.051)	1.001 (0.999, 1.002)
Clinical Pregnancy → Live births	1.014 (1.001, 1.027)	1.001 (0.997, 1.004)	1.007 (0.995, 1.019)	1.010 (1.002, 1.019)	1.001 (0.999, 1.001)	1.041 (0.965, 1.122)	1.001 (0.999, 1.002)

Note: CI, confidence interval; AMH, Anti-mullerian hormone; AFC, Antral Follicle Count; FSH, Follicle Stimulating Hormone; LH, Luteinizing Hormone; E2, Estradiol; P, Progesterone; TSH, Thyroid-Stimulating Hormone.

^a^Model 1: analysis adjusted for Female age and ethnicity.

^b^Model 2: further adjusted for female education, male smoking, body mass index, total bilirubin, direct bilirubin, blood glucose, number of high-quality cleavage-stage embryos, number of high-Quality embryos transferred, types of infertility, endometrial thickness, treatment programmes, female health status.

### Using multistate modelling to investigate the relationship between reproductive endocrine hormones and progression to assisted conception outcomes in infertile patients

**Fig 3:**
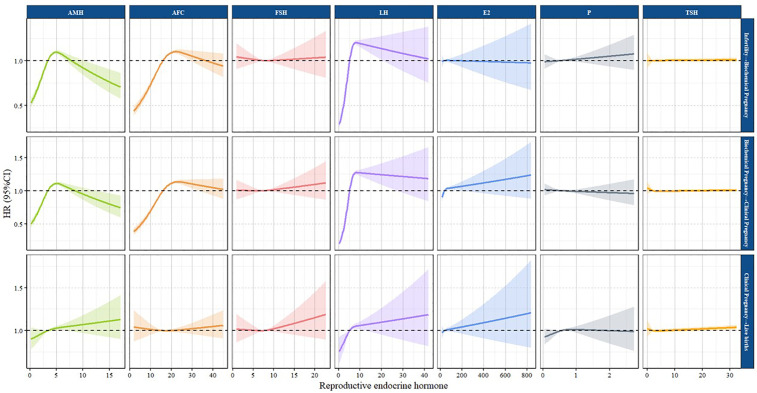
Using a multistate model (N = 12,674), we examined the associations between reproductive endocrine hormones and transitions among three states in infertile patients. Hazard ratios are represented by bold lines, with 95% confidence intervals indicated by shaded areas. In the eight figures depicting the relationships of AMH and LH with the three state transitions (from infertility to biochemical pregnancy, biochemical pregnancy to clinical pregnancy, and clinical pregnancy to live birth) and AFC with the transitions from infertility to biochemical pregnancy and from biochemical pregnancy to clinical pregnancy, the P-values for non-linearity tests were all less than 0.05. Additionally, the P-values for non-linearity tests in the remaining figures were all greater than 0.05. Note: AMH, Anti-mullerian hormone; AFC, Antral Follicle Count; FSH, Follicle Stimulating Hormone; LH, Luteinizing Hormone; E2, Estradiol; P, Progesterone; TSH, Thyroid-Stimulating Hormone.

The dose-response relationship curves derived from the multi-state regression model revealed significant non-linear associations between AMH and LH and the three state transitions (from infertility to biochemical pregnancy, biochemical pregnancy to clinical pregnancy, and clinical pregnancy to live birth), as well as between AFC and the transitions from infertility to biochemical pregnancy and from biochemical pregnancy to clinical pregnancy (all P for non-linearity < 0.05), suggesting that the relationships between these indicators and state transitions are non-linear and may involve threshold effects. In contrast, other indicators exhibited P for non-linearity values greater than 0.05 (Fig 3). Table 3 presents the estimated relationships between reproductive endocrine hormones and the three assisted reproductive outcome transitions. Increases in AMH and LH were associated with higher positive rates of the three assisted reproductive outcome transitions, while AFC was associated with higher positive rates for the transitions from infertility to biochemical pregnancy and from biochemical pregnancy to clinical pregnancy. E2 was associated with a higher positive rate for the transition from biochemical pregnancy to clinical pregnancy. These associations remained significant after adjusting for basic demographic variables, clinical indicators, and laboratory test results. The associations of AMH and LH with the three assisted reproductive outcome transitions were stronger than those of AFC and E2. Specifically, each 1-unit increase in AMH and LH was significantly associated with an elevated incidence of patients progressing from infertile status to a positive biochemical pregnancy, from a positive biochemical pregnancy to a positive clinical pregnancy, and from a positive clinical pregnancy to a live birth, with HRs of 1.027 (95%CI: 1.018, 1.037), 1.013 (95%CI: 1.002 1.024), 1.014 (95% CI: 1.001, 1.026), 1.025 (95% CI: 1.021, 1.029), 1.026 (95% CI: 1.020, 1.032), and 1.007 (95% CI: 1.002, 1.015).Each 1-unit increase in AFC was associated with the patient’s progression from infertile status to biochemical pregnancy positivity, progression from biochemical pregnancy positivity to clinical pregnancy positivity, and progression from clinical pregnancy positivity to live birth had HRs of 1.013 (95% CI: 1.010, 1.017), 1.008 (95% CI: 1.005, 1.012), and 1.001 (95% CI: 0.997, 1.004), respectively. Each 1-unit increase in E2 was associated with a HR of 1.001 (95% CI: 1.001, 1.002) for the transition from biochemical pregnancy to clinical pregnancy.

**Table 3:** Hazard ratios (95% CI) associated with each 1-unit increase in reproductive endocrine hormones with biochemical pregnancy, clinical pregnancy, and subsequent live birth, calculated using a multistate model (N = 12,674).

Model	AMH	AFC	FSH	LH	E2	P	TSH
Model 1^a^							
Infertility → Biochemical Pregnancy	1.028 (1.018, 1.038)	1.014 (1.011, 1.017)	0.996 (0.986, 1.006)	1.025 (1.021, 1.029)	0.999 (0.999, 1.001)	1.011 (0.951, 1.074)	1.001 (0.999, 1.001)
Biochemical Pregnancy → Clinical Pregnancy	1.013 (1.002, 1.024)	1.008 (1.005, 1.011)	1.006 (0.995, 1.017)	1.026 (1.020, 1.032)	1.001 (1.001, 1.002)	0.957 (0.894, 1.025)	1.001 (0.999, 1.001)
Clinical Pregnancy → Live births	1.014 (1.002, 1.027)	1.002 (0.998, 1.006)	1.002 (0.990, 1.013)	1.007 (1.002, 1.017)	1.001 (0.999, 1.001)	1.042 (0.967, 1.124)	1.001 (0.999, 1.002)
Model 2^b^							
Infertility → Biochemical Pregnancy	1.027 (1.018, 1.037)	1.013 (1.010, 1.017)	0.997 (0.987, 1.004)	1.025 (1.021, 1.029)	0.999 (0.999, 1.001)	1.018 (0.958, 1.082)	1.001 (0.999, 1.001)
Biochemical Pregnancy → Clinical Pregnancy	1.013 (1.002, 1.024)	1.008 (1.005, 1.012)	1.006 (0.995, 1.017)	1.026 (1.020, 1.032)	1.001 (1.001, 1.002)	0.959 (0.896, 1.027)	1.001 (0.999, 1.001)
Clinical Pregnancy → Live births	1.014 (1.001, 1.026)	1.001 (0.997, 1.004)	1.002 (0.990, 1.013)	1.007 (1.002, 1.015)	1.001 (0.999, 1.001)	1.051 (0.974, 1.134)	1.001 (0.999, 1.002)

Note: CI, confidence interval; AMH, Anti-mullerian hormone; AFC, Antral Follicle Count; FSH, Follicle Stimulating Hormone; LH, Luteinizing Hormone; E2, Estradiol; P, Progesterone; TSH, Thyroid-Stimulating Hormone.

^a^Model 1: analysis adjusted for Female age and ethnicity.

^b^Model 2: further adjusted for female education, male smoking, body mass index, total bilirubin, direct bilirubin, blood glucose, number of high-quality cleavage-stage embryos, number of high-Quality embryos transferred, types of infertility, endometrial thickness, treatment programmes, female health status.

### Sensitivity analysis

The associations between reproductive endocrine hormones and the progression of three assisted reproductive outcomes in infertile patients remained stable across various sensitivity analyses. First, after excluding 294 participants with more than 10 years of infertility, the results indicate that the association between reproductive parameters and the progression of the three pregnancy outcomes has not undergone any substantial change (S1 Table). Second, subgroup analyses further showed that the direction of the association between exposure and the three assisted reproduction outcomes remained consistent regardless of whether the pregnant woman had a normal BMI (S2 Table)

## Discussion

This cohort study, encompassing 12,674 patients undergoing fresh embryo transfer, categorized outcomes as no pregnancy, biochemical pregnancy, clinical pregnancy, and live birth. Our analysis revealed that female age, female educational level, female BMI, serum total bilirubin, serum direct bilirubin, fasting blood glucose, number of good-quality cleavage-stage embryos, number of good-quality embryos transferred, infertility etiology, endometrial thickness on the day of transfer, AMH, AFC, basal FSH, basal LH, basal E2, basal P, TSH, and the ovarian stimulation protocol all influenced the outcomes of fresh embryo transfer. 2024 study by Siladitya Bhattacharya identified key predictors of IVF treatment success and assessed their impact on live birth rates, pinpointing 11 predictors: age, duration of infertility, ethnicity, body mass index, antral follicle count, previous pregnancy history, cause of infertility, sperm parameters, number of oocytes collected, morphology of transferred embryos, and day of embryo transfer [19]. This aligns partially with our findings regarding common influencing factors (including age, BMI, AFC, infertility etiology, and morphology of transferred embryos). However, our study additionally found that sex hormones also impacted pregnancy outcomes.

Utilizing a multistate regression model in what is, to date, the largest cohort of Chinese infertile patients undergoing fresh-cycle embryo transfer, this study thoroughly investigated the association between reproductive endocrine hormones (AMH, AFC, FSH, LH, E2, P, TSH) and the continuous transition process of Assisted Reproductive Technology (ART) outcomes (from infertility to biochemical pregnancy, from biochemical pregnancy to clinical pregnancy, and from clinical pregnancy to live birth). Our findings reveal that AMH and LH exhibit significant positive associations with all stages of ART outcomes, including eventual live birth, and these relationships demonstrate non-linear characteristics. AFC showed significant predictive value for early pregnancy stages (biochemical and clinical pregnancy) but was not significantly associated with live birth.

### Association of AMH with pregnancy outcomes

AMH, a crucial marker of ovarian reserve [20–22], demonstrated a significant impact on the transition from infertility to biochemical pregnancy, clinical pregnancy, and live birth in this study. In Model 1 and Model 2, for each one-unit increase in AMH levels, the hazard ratios (HRs) for biochemical pregnancy were 1.028 (95% CI: 1.018–1.038) and 1.027 (95% CI: 1.018–1.037), respectively, indicating a positive correlation between elevated AMH levels and the occurrence of biochemical pregnancy. This is consistent with previous research [23–25], where higher AMH levels, reflecting better ovarian reserve, are associated with an increased chance of biochemical pregnancy [23,24,26,27]. However, the association between AMH and live birth rates (LBRs) has been debated. Some studies suggest a positive correlation [25,28], while others indicate a decline in LBRs in patients with extremely high AMH levels, potentially due to an increased risk of ovarian hyperstimulation syndrome (OHSS) leading to fresh embryo transfer cancellation [29,30]. Our study, using a multistate model in a large cohort, further confirms the positive predictive role of AMH for live birth, even after adjusting for age and multiple clinical confounders. This may suggest that, after excluding patients whose transfers were cancelled due to severe OHSS risk (this study focused on fresh-cycle transfer patients but could not directly distinguish if a “freeze-all” was due to OHSS risk) and within our age range (20–40 years), elevated AMH generally remains beneficial for pregnancy outcomes. The non-linear relationship of AMH suggests an optimal range, with our study identifying an optimal AMH threshold of 4–8 ng/mL. Beyond this range, its benefits may plateau or even decline, echoing the sentiment of Acharya et al. That “higher AMH is not always better” [29]. Very low AMH levels (typically <1.0–1.2 ng/mL, or even lower) indicate diminished ovarian reserve, fewer oocytes, and poor response to stimulation, leading to reduced oocyte yield, fertilization rates, and good-quality embryo formation, ultimately impacting pregnancy and live birth rates [21,27,31]. Within the optimal AMH range, ovarian responsiveness is good, yielding an adequate number of oocytes and high-quality embryos, thereby maximizing the chances of pregnancy and live birth. Although higher AMH generally indicates abundant ovarian reserve [32], when AMH levels are excessively high (e.g., commonly seen in PCOS patients, > 10 ng/mL or even higher), its benefit for LBR may diminish [29,30]. Potential reasons include an increased risk of OHSS, as patients with high anti-Müllerian hormone (AMH) levels are more sensitive to stimulation drugs and thus more susceptible to OHSS. To avoid OHSS, clinicians may opt for a “freeze-all” strategy, cancelling the fresh-cycle transfer. Although this study focused on fresh cycles, the OHSS risk associated with high AMH might complicate clinical management for some patients (even those undergoing fresh transfer), thereby affecting final live birth [33]. Oocyte quality issues: Despite a high oocyte yield, oocyte quality in patients with extremely high AMH (especially PCOS with high AMH) may be compromised, manifested as reduced maturation rates, fertilization rates, or developmental potential of embryos [27].Abnormal endocrine environment: High AMH levels may be accompanied by other endocrine disorders, such as hyperandrogenism or insulin resistance, which themselves could adversely affect endometrial receptivity or embryo implantation [19,34–36].

### Association of AFC with pregnancy outcomes

Our results showed that for each one-unit increase in AFC, the risk of transitioning from infertility to biochemical pregnancy and from biochemical pregnancy to clinical pregnancy significantly increased (HRs of 1.014 and 1.008, respectively), with a non-linear relationship. However, unlike AMH, AFC was not significantly associated with the transition from clinical pregnancy to live birth (HR = 1.002, 95% CI: 0.998, 1.006). This finding aligns with AFC’s role as an ovarian reserve marker, effectively predicting oocyte yield and early pregnancy establishment [21,32,37]. The lack of significant prediction by AFC for the transition from clinical pregnancy to live birth may imply that while follicle count plays a key role in successful implantation and early development, factors such as embryo quality, endometrial receptivity, and other unknown variables might be more crucial for maintaining pregnancy through mid and late gestation (to live birth) [37,38]. The study by Sahu et al. also found AFC to have low discriminatory power for predicting miscarriage [37], which is similar to our finding of AFC’s non-significant predictive efficacy in the clinical pregnancy to live birth stage.

### Association of LH with pregnancy outcomes

This study, for the first time on a large scale, reveals that basal LH levels exhibit a significant positive association with all three stages of ART pregnancy transition (infertility to biochemical pregnancy, biochemical pregnancy to clinical pregnancy, and clinical pregnancy to live birth), and this relationship is non-linear. This finding is particularly important as it challenges the conventional perception that generally associates high basal LH with adverse pregnancy outcomes. Contrast with traditional understanding: Traditionally, especially in PCOS patients, excessively high basal LH has been thought to potentially lead to abnormal follicular development, reduced oocyte quality, and even increased miscarriage risk [39,40]. However, our findings are similar to observations by Sun et al. in PCOS patients [41], suggesting that “high” basal LH within a certain range does not necessarily lead to adverse outcomes and may even be associated with better oocyte and embryo yield. Possible implications of LH’s non-linear association, Extremely low LH: If basal LH levels are too low (e.g., in patients with GnRH deficiency or hypothalamic amenorrhea), they may not provide sufficient physiological LH pulses to support normal follicular development and function, thereby affecting pregnancy outcomes [42–45]. Moderate to slightly elevated LH: Within a physiological or slightly elevated LH range, LH may exert its positive role as a key regulator of follicular development and oocyte maturation, promoting follicle growth, estrogen synthesis, and oocyte maturation, thereby improving pregnancy and live birth rates. This “slightly elevated” LH might help optimize ovarian response to stimulation drugs or reflect a more favorable endocrine state for pregnancy in some individuals [41,46–49]. Potential impact of extremely high LH: Although the overall trend in our study was a positive association, the non-linearity might capture a scenario where, at extremely high LH levels, the benefits begin to plateau, or in extreme cases, such as those accompanied by severe hyperandrogenism or significant follicular development abnormalities, negative effects might emerge [39,40]. This may warrant more detailed analysis, such as subgroup analysis by specific LH quantiles.

### Association of FSH, E2, P, and TSH with pregnancy outcomes

Hormones such as FSH, E2, P, and TSH also play crucial roles in the reproductive process. In our study, elevated FSH levels were negatively associated with the transition from infertility to biochemical pregnancy, which may be related to elevated FSH indicating diminished ovarian reserve. Elevated E2 levels were positively associated with the occurrence of biochemical pregnancy, but the relationship with LBR was unclear. This could be because elevated E2 levels might indicate both a good ovarian response to stimulation and an increased risk of OHSS. Elevated P levels showed no significant association with the transition to biochemical pregnancy, clinical pregnancy, or live birth, which might relate to progesterone’s role in pregnancy maintenance. Elevated TSH levels also showed no significant association with these transitions. This could be because patients with thyroid dysfunction would have received appropriate specialist thyroid intervention to optimize thyroid function for conception before commencing IVF.

## Conclusion

In conclusion, this study confirms that baseline anti-Müllerian hormone (AMH) and luteinizing hormone (LH) are robust and independent biomarkers for predicting the entire continuum from infertility to live birth in first fresh-cycle IVF. Furthermore, our data indicate that the choice of treatment protocol is also significantly associated with the outcome, with the long protocol being linked to higher live birth rates. Integrating these findings, our study suggests that for patients with baseline AMH levels between 4–8 ng/mL and LH levels between 4–10 mIU/mL, employing a long stimulation protocol may represent the optimal strategy to maximize the live birth rate in fresh embryo transfer cycles. The reliability of these conclusions is bolstered by the study’s main strengths: its large sample size and the use of an advanced multi-state model, which comprehensively tracked outcomes to live birth while adjusting for key confounders like treatment protocol. Nevertheless, some limitations should be acknowledged. As a retrospective cohort study, the potential for unmeasured confounders remains. Additionally, our focus on fresh-cycle transfers means the applicability of these findings to “freeze-all” strategies requires further investigation.

## Supporting information

S1 Table Hazard ratios (95% CI) associated with each 1-unit increase in baseline endocrine factors with biochemical pregnancy, clinical pregnancy, and subsequent live birth, calculated using a multistate model (N = 12,380). (DOCX)

S2 Table Hazard ratios (95% CI) associated with each 1-unit increase in reproductive endocrine hormones with biochemical pregnancy, clinical pregnancy, and subsequent live birth, calculated using a multistate model (N = 12,674). (DOCX)
