# Supplementary material for: Baseline endocrine factors influencing live birth outcomes in Chinese infertile women undergoing their first fresh IVF cycle: A multistate model-based cohort study
Source: PLoS One. 2026 Jun 5;21(6):e0349394. doi: 10.1371/journal.pone.0349394 (PMC13240884; doi:10.1371/journal.pone.0349394)
Supplement: S1 Table — (DOCX) [file pone.0349394.s001.docx]

**Table S1.** Hazard ratios (95% CI) associated with each 1-unit increase in baseline endocrine factors with biochemical pregnancy, clinical pregnancy, and subsequent live birth, calculated using a multistate model (N = 12,380).

| Model | AMH | AFC | FSH | LH | E2 | P | TSH |
| --- | --- | --- | --- | --- | --- | --- | --- |
| Model 1^a^ |  |  |  |  |  |  |  |
| Infertility →  Biochemical Pregnancy | 1.028  (1.017, 1.039) | 1.016  (1.011, 1.019) | 0.997  (0.986, 1.007) | 1.026  (1.021, 1.030) | 0.998  (0.996, 1.001) | 1.010  (0.949, 1.075) | 1.001  (0.999, 1.003) |
| Biochemical Pregnancy →  Clinical Pregnancy | 1.014  (1.001, 1.024) | 1.009  (1.005, 1.012) | 1.006  (0.995, 1.017) | 1.028  (1.018, 1.039) | 1.002  (1.001, 1.003) | 0.958  (0.894, 1.026) | 1.001  (0.999, 1.003) |
| Clinical Pregnancy →  Live births | 1.015  (1.002, 1.028) | 1.003  (0.998, 1.009) | 1.003  (0.990, 1.014) | 1.007  (1.002, 1.017) | 1.001  (0.999, 1.002) | 1.043  (0.966, 1.125) | 1.001  (0.999, 1.002) |
| Model 2^b^ |  |  |  |  |  |  |  |
| Infertility →  Biochemical Pregnancy | 1.028  (1.018, 1.039) | 1.014  (1.010, 1.018) | 0.998  (0.987, 1.005) | 1.025  (1.020, 1.030) | 0.999  (0.998, 1.001) | 1.019  (0.957, 1.083) | 1.001  (0.999, 1.003) |
| Biochemical Pregnancy →  Clinical Pregnancy | 1.012  (1.001, 1.024) | 1.009  (1.005, 1.013) | 1.008  (0.998, 1.019) | 1.027  (1.022, 1.033) | 1.002  (1.001, 1.003) | 0.960  (0.896, 1.028) | 1.001  (0.999, 1.002) |
| Clinical Pregnancy →  Live births | 1.015  (1.001, 1.029) | 1.002  (0.997, 1.007) | 1.003  (0.993, 1.014) | 1.007  (1.001, 1.014) | 1.001  (0.999, 1.002) | 1.061  (0.974, 1.144) | 1.001  (0.999, 1.002) |

Note: CI, confidence interval; AMH, Anti-mullerian hormone; AFC, Antral Follicle Count; FSH, Follicle Stimulating Hormone; LH, Luteinizing Hormone; E2, Estradiol; P, Progesterone; TSH, Thyroid-Stimulating Hormone.

^a^ Model 1: analysis adjusted for Female age and ethnicity.

^b^ Model 2: further adjusted for female education, male smoking, body mass index, total bilirubin, direct bilirubin, blood glucose, number of high-quality cleavage-stage embryos, number of high-Quality embryos transferred, types of infertility, endometrial thickness, treatment programmes, female health status.
