# Supplementary material for: Baseline endocrine factors influencing live birth outcomes in Chinese infertile women undergoing their first fresh IVF cycle: A multistate model-based cohort study
Source: PLoS One. 2026 Jun 5;21(6):e0349394. doi: 10.1371/journal.pone.0349394 (PMC13240884; doi:10.1371/journal.pone.0349394)
Supplement: S2 Table — (DOCX) [file pone.0349394.s002.docx]

**Table S2.** Hazard ratios (95% CI) associated with each 1-unit increase in reproductive endocrine hormones with biochemical pregnancy, clinical pregnancy, and subsequent live birth, calculated using a multistate model (N = 12,674).

| Model | AMH | AFC | FSH | LH | E2 | P | TSH |
| --- | --- | --- | --- | --- | --- | --- | --- |
| **Normal weight (9421)** |  |  |  |  |  |  |  |
| Infertility →  Biochemical Pregnancy | 1.029  (1.016, 1.039) | 1.015  (1.010, 1.021) | 0.998  (0.987, 1.010) | 1.026  (1.021, 1.030) | 0.998  (0.996, 1.001) | 1.019  (0.958, 1.082) | 1.001  (0.998, 1.004) |
| Biochemical Pregnancy →  Clinical Pregnancy | 1.017  (1.002, 1.034) | 1.010  (1.002, 1.019) | 1.009  (0.992, 1.019) | 1.027  (1.020, 1.035) | 1.001  (1.001, 1.002) | 0.960  (0.896, 1.029) | 1.001  (0.999, 1.003) |
| Clinical Pregnancy →  Live births | 1.016  (1.001, 1.032) | 1.002  (0.999, 1.005) | 1.003  (0.990, 1.017) | 1.011  (1.002, 1.020) | 1.001  (0.999, 1.001) | 1.053  (0.972, 1.136) | 1.001  (0.999, 1.002) |
| **Overweight or obese (3253)** |  |  |  |  |  |  |  |
| Infertility →  Biochemical Pregnancy | 1.030  (1.013, 1.048) | 1.017  (1.011, 1.023) | 0.998  (0.987, 1.010) | 1.027  (1.020, 1.035) | 0.999  (0.997, 1.001) | 1.020  (0.958, 1.082) | 1.002  (0.998, 1.006) |
| Biochemical Pregnancy →  Clinical Pregnancy | 1.015  (1.005, 1.026) | 1.013  (1.007, 1.020) | 1.009  (0.992, 1.019) | 1.027  (1.018, 1.036) | 1.003  (1.001, 1.005) | 0.962  (0.896, 1.029) | 1.002  (0.997, 1.007) |
| Clinical Pregnancy →  Live births | 1.013  (1.002, 1.025) | 1.002  (0.999, 1.005) | 1.003  (0.991, 1.016) | 1.012  (1.004, 1.020) | 1.003  (0.995, 1.011) | 1.051  (0.969, 1.138) | 1.002  (0.998, 1.007) |

Note: CI, confidence interval; AMH, Anti-mullerian hormone; AFC, Antral Follicle Count; FSH, Follicle Stimulating Hormone; LH, Luteinizing Hormone; E2, Estradiol; P, Progesterone; TSH, Thyroid-Stimulating Hormone.

Analysis adjusted for female age, female ethnicity, female education, male smoking, body mass index, total bilirubin, direct bilirubin, blood glucose, number of high-quality cleavage-stage embryos, number of high-Quality embryos transferred, types of infertility, endometrial thickness, treatment programmes, female health status.
